# Sub-Acute Feeding Study of Saxitoxin to Mice Confirms the Effectiveness of Current Regulatory Limits for Paralytic Shellfish Toxins

**DOI:** 10.3390/toxins13090627

**Published:** 2021-09-07

**Authors:** Sarah C. Finch, Nicola G. Webb, Michael J. Boundy, D. Tim Harwood, John S. Munday, Jan M. Sprosen, Vanessa M. Cave, Ric B. Broadhurst, Jeane Nicolas

**Affiliations:** 1 AgResearch Ltd. Ruakura Research Centre, Private Bag 3123, Hamilton 3240, New Zealand; nikki.webb@agresearch.co.nz (N.G.W.); jan.sprosen@agresearch.co.nz (J.M.S.); vanessa.cave@agresearch.co.nz (V.M.C.); ric.broadhurst@agresearch.co.nz (R.B.B.); 2 Cawthron Institute, Private Bag 2, Nelson 7042, New Zealand; Michael.Boundy@cawthron.org.nz (M.J.B.); Tim.Harwood@cawthron.org.nz (D.T.H.); 3 Department of Pathobiology, School of Veterinary Science, Massey University, Private Bag 11 222, Palmerston North 4442, New Zealand; J.Munday@massey.ac.nz; 4 Ministry for Primary Industries–Manatu Ahu Matua, P.O. Box 2526, Wellington 6021, New Zealand; Jeane.Nicolas@mpi.govt.nz

**Keywords:** saxitoxin, feeding study, toxicology, paralytic shellfish toxins

## Abstract

Regulatory limits for shellfish toxins are required to protect human health. Often these limits are set using only acute toxicity data, which is significant, as in some communities, shellfish makes up a large proportion of their daily diet and can be contaminated with paralytic shellfish toxins (PSTs) for several months. In the current study, feeding protocols were developed to mimic human feeding behaviour and diets containing three dose rates of saxitoxin dihydrochloride (STX.2HCl) were fed to mice for 21 days. This yielded STX.2HCl dose rates of up to 730 µg/kg bw/day with no effects on food consumption, growth, blood pressure, heart rate, motor coordination, grip strength, blood chemistry, haematology, organ weights or tissue histology. Using the 100-fold safety factor to extrapolate from animals to humans yields a dose rate of 7.3 µg/kg bw/day, which is well above the current acute reference dose (ARfD) of 0.5 µg STX.2HCl eq/kg bw proposed by the European Food Safety Authority. Furthermore, to reach the dose rate of 7.3 µg/kg bw, a 60 or 70 kg human would have to consume 540 or 630 g of shellfish contaminated with PSTs at the current regulatory limit (800 µg/kg shellfish flesh), respectively. The current regulatory limit for PSTs therefore seems appropriate.

## 1. Introduction

Paralytic shellfish poisoning (PSP) is induced by the ingestion of shellfish contaminated with paralytic shellfish toxins (PSTs). These toxins are produced by the marine dinoflagellates of the genera *Alexandruim*, *Gymnodinium* and *Pyrodinium* [1,2,3], and are accumulated by filter-feeding shellfish. The major PST is saxitoxin (STX), although over fifty structurally related analogues make up this toxin class [4]. PSP is characterised by tingling and numbness around the lips, incoordination and muscle weakness, as well as neurological symptoms such as headaches [5]. In severe cases, muscular paralysis will be marked and respiratory paralysis can result in death. This intoxication is not location specific and throughout history PSP outbreaks have been regularly reported worldwide [6,7]. The first such report was documented in a ship captain’s diary in 1793 when five crew members became ill after eating mussels harvested off the coast of British Columbia which resulted in one death [8]. In Alaska, the first case of PSP was in 1799, and between 1973 and 1994, there were 54 outbreaks resulting in 117 people falling ill. Of those affected, one person died, four required intubation and 29 required emergency treatment [9]. In order to protect human health, a regulatory limit for PSTs has been adopted by many countries. Currently the regulatory limit for the European Union (EU) is set at 800 µg STX.2HCl eq/kg shellfish flesh (regulation (EC) No 853/2004) [10]. The same limit was adopted at the twenty-eighth session of the Codex Committee on Fish and Fishery Products (CCFFP) in 2006 [11], resulting in the development of the Standard for Live and Raw Bivalve Molluscs (CODEXSTAN 292-2008, rev. 2015) [12]. This standard is used in many countries, including New Zealand. Because STX hydrate (the free base) is of low stability, the STX limit is expressed as saxitoxin dihydrochloride (STX.2HCl). It is of vital importance that whenever PST mass concentrations are mentioned, it is specified whether this refers to the salt (STX.2HCl) or to the free base (STX hydrate). This information is needed to interpret results and to allow the comparison of concentrations quoted in different studies [13]. The regulatory limit is also expressed in terms of STX equivalents (STX.2HCl eq) as the PSTs include more than just STX, and other derivatives may simultaneously be present in the shellfish ingested by humans. To accommodate this, the toxicity data of each analogue is compared to that of STX on a molar basis to yield a toxicity equivalence factor (TEF). Using the TEF values, the concentrations of each analogue, as determined by analytical methods, can be converted into STX.2HCl equivalents such that the overall toxicity of a given sample can be evaluated against the regulatory limit.

Estimating the quantity of PSTs that have been responsible for reported cases of human illness is very difficult as a number of different pieces of information are required including an accurate determination of the amount of toxin in the food (STX.2HCl eq), the quantity of contaminated shellfish eaten, and the bodyweight of the person. The quantity of shellfish eaten is often not known and it is rare for the weight of the patient to be reported in the literature. Furthermore, there is often a time lag between the consumption of contaminated shellfish and the diagnosis of PSP, meaning that remnant food is rarely available for analysis. While shellfish can be harvested from the affected area, this is not necessarily representative of the food ingested and it has been demonstrated that the concentrations of PSP toxins can change very quickly in shellfish [14]. In addition, the effects of different cooking processes on PST concentrations are uncertain and whether broth or cooking liquid is consumed or discarded adds another layer of complexity [15,16].

By considering the available data on human PSP cases, the European Food Safety Authority (EFSA) determined that the quantity of STX which could be consumed in a 24 h period without adverse effect, the acute reference dose (ARfD), was 0.5 µg STX.2HCl eq/kg bw [7]. To relate this to the quantities which could be ingested by consumers of shellfish, three pieces of information are required: the STX concentration in the shellfish, the quantity of shellfish consumed (portion size), and the bodyweight of the consumer. In calculations of risk, the concentration of STX in shellfish is assumed to be at the current NZ/Codex/EU regulatory limit (800 µg STX.2HCl eq/kg shellfish flesh). Understandably, portion size will vary greatly throughout the population, making it difficult to select an appropriate value. A large portion size of 400 g has been proposed by EFSA which represents the 95th percentile of shellfish consumption in Germany and the Netherlands [17]. However, this value appears high as the 97.5th percentile for the portion size of shellfish eaten by adults is 133 g in Japan, 181 g in Australia, 225 g in the USA and 263 g in New Zealand, and a portion size of 250 g would cover 97.5% of the consumers of most countries for which data are available [8]. Although the bodyweight of adults is also highly variable, it is imperative that risk assessments are done using conservative estimates, thus ensuring that the majority of the population is covered. EFSA has used an adult bodyweight of 60 kg [7], but a further EFSA committee has argued that a standard bodyweight of 70 kg would be more appropriate [18]. Looking at the range of possible portion sizes of shellfish in particular, but also bodyweights, illustrates that the estimate of STX which could be ingested by a human varies widely. Using the worst-case scenario of shellfish at the regulatory limit (800 µg STX.2HCl eq/kg shellfish flesh), a portion size of 400 g and a 60 kg adult, the dose rate consumed would be 5.3 µg STX.2HCl eq/kg bw. However, consumption of shellfish at the regulatory limit with a portion size of 250 g by a 70 kg adult would yield a dose rate of 2.9 µg STX.2HCl eq/kg bw. Although significantly different, both of these figures are above the ARfD of 0.5 µg STX.2HCl eq/kg bw. This difference was acknowledged by EFSA who concluded “there is a concern for the health for the consumer at the present regulatory limit” [7].

Given the uncertainties, generating regulatory limits using human cases of poisoning is difficult and as an alternative animal models can be used. Oral toxicity is the most relevant route of administration and, when available, these data are used in preference to toxicity determined by intraperitoneal injection (i.p.) [19]. To extrapolate from animal data to human, uncertainty factors are applied to animal toxicity data. Those generally applied are a ten-fold safety factor to allow for the species difference, and a further ten-fold safety factor to allow for possible susceptibility variations within a human population [20]. An acute no observable adverse effect level (NOAEL) for STX of 473 µg STX.2HCl eq/kg bw has been determined in mice using oral administration [21]. When the combined safety factors of 100 are applied to this figure, the concentration which would be expected to induce no adverse effects in humans is 4.7 µg STX.2HCl eq/kg bw. This dose rate is much higher than the ARfD suggested by EFSA (0.5 µg STX.2HCl eq/kg bw). Comparing the Health Based Guidance Value (HBGV) to the current STX.2HCl regulatory limit of 800 µg STX.2HCl eq/kg shellfish flesh is difficult because this is dependent on an estimate of portion size and human bodyweight.

While there is still debate around the validity of the current regulatory limit for PSTs, of further concern is the fact that people who eat shellfish often do so on a regular basis, and for some communities, shellfish constitutes a high proportion of their diet. Since algal blooms can be present for several months, shellfish may be contaminated with PSTs for an extended time [22,23,24]. Despite this, as noted by the FAO/IOC/WHO (2004) Committee, no repeated oral toxicity studies have been performed to assess whether STX has a cumulative effect or whether more subtle indications of toxicity may arise with regular sub-lethal doses [8]. To fill this significant knowledge gap, a sub-acute study with daily dosing of STX.2HCl to mice was performed. Feeding protocols using meal times were developed in order to more closely represent average human feeding behaviour.

## 2. Results

### 2.1. Development of Experimental Protocols

#### 2.1.1. Development of Suitable Diet

Previous mouse feeding studies have been conducted by incorporating the test ingredient, such as ground endophyte-infected ryegrass seed [25] or the endophyte-expressed metabolite chanoclavine [26] with ground mouse food. In these studies, the diet portions weighed approximately 7 g and the compounds were found to be homogenous in the diet. However, using the same protocol, STX was found to be unevenly distributed in the diet with the STX.2HCl concentration 24–40% higher at the edges of the diet portion in comparison to the middle. This difference is likely due to the high water solubility of STX which allows it to be redistributed in the diet during drying, a process which would not be possible for the seed or highly lipophilic compound used in the other studies. Modifications to the protocol where smaller portions of diet (approximately 1 g each) were prepared using a lower drying temperature for a shorter time rectified this issue and STX.2HCl was confirmed to be homogenously distributed in this diet.

The stability of STX.2HCl in laced diet was assessed by storing individual portions (1 g) for a number of days in the fridge (4 °C) or at room temperature (20 °C). This showed that STX was stable in the diet for up to five days at both temperatures (Table 1). Since fresh diet was prepared every 2–4 days during the feeding study, the stability of STX in the mouse diet was judged to be adequate.

#### 2.1.2. Establishment of a Suitable STX Dose Rate

Many studies on shellfish toxins using mice have successfully utilised a method of oral administration whereby the test toxin is mixed with a very small amount of cream cheese [27,28]. After training with unlaced cream cheese for a few days prior, mice will consume this laced cream cheese within 30 s. Using this method, an acute oral dose rate of 400 µg STX.2HCl/kg bw induced mild toxic effects in one out of three mice, so it was anticipated that this could be an appropriate dose rate for the high STX treatment group in the feeding study. However, it was found that mice with unlimited access to STX-laced diet, delivering a daily dose of 476 µg STX.2HCl/kg bw for two weeks, showed no adverse effects. Furthermore, mice with unlimited access to STX-laced diet to deliver a daily dose rate of 620 µg STX.2HCl/kg bw for four days were also unaffected. This demonstrated that the toxicity of STX could be influenced by the feeding protocols used in the study, highlighting the importance of using a feeding protocol that is relevant to humans. In contrast to the human feeding behaviour of meal times, the feeding behaviour of mice is that of continuous grazing. To develop a relevant feeding protocol, pairs of individually caged mice were offered either unrestricted access to control diet (no STX) or access to the same food between only 9–11 a.m. and 3–5 p.m., with food consumption being measured in both groups. This showed that for two days, mice with limited feeding times had a low daily food intake (0.029 and 0.176 g food/g bw on days 1 and 2, respectively) compared to those with unlimited access to food (0.314 and 0.296 g food/g bw on days 1 and 2, respectively). However, by day 3, the two groups had an equivalent total food consumption (0.256 compared to 0.240 g food/g bw, for unlimited and limited, respectively). For mice fed between 9–11 a.m. and 3–5 p.m., it was observed that, at the most, they ate for only half of their 2 h feeding period before going to sleep. Meal times of a 1 h duration were therefore chosen for the feeding study.

Since no toxicity was observed on feeding STX.2HCl in laced diet, the possible effect of the matrix was investigated. A mouse with unrestricted access to control diet dosed with STX.2HCl in cream cheese to deliver a 400 µg STX.2HCl/kg bw dose showed a hunched posture and splayed back legs 2 h post-dosing, symptoms characteristic of STX toxicity. Using the meal time feeding protocol, a further two mice were taken, with one fed a STX.2HCl-laced diet and the other fed a control diet, but half way through each feeding period STX.2HCl laced cream cheese was offered, and consumed by the mouse within 30 s. Each of these latter two mice ingested 400 µg STX.2HCl/kg bw at each feeding period (800 µg/kg bw daily total) and neither showed any adverse effects. This demonstrates that the lack of STX.2HCl toxicity is the same whether fed via mouse food diet laced with STX or in laced cream cheese, eliminating the possibility of a matrix effect.

### 2.2. Results of the 21-Day Feeding Study

#### 2.2.1. Diet Analysis

Preliminary work and palatability studies allowed the STX.2HCl concentrations for the feeding study to be chosen. Laced diet from each treatment group was analysed to ensure that the STX.2HCl concentrations were as expected. A comparison between the theoretical and actual concentrations of STX.2HCl in each of the three treatment groups showed this to be the case (Table 2).

#### 2.2.2. Dose Rates

By combining the daily food intake and bodyweight data for each individual animal, the dose rate of STX.2HCl consumed each day could be calculated, thereby allowing the mean daily dose rate of STX.2HCl to be determined for each treatment group. The dose rates showed good consistency throughout the 21-day feeding period with means (± standard error) of 253 ± 5 and 248 ± 5 µg/kg bw/day for female and male mice fed with low-dose STX-laced diet, 494 ± 9 and 486 ± 8 µg/kg bw/day for female and male mice fed mid-dose STX-laced diet, and 730 ± 13 and 699 ± 11 µg/kg bw/day for female and male mice fed high-dose STX-laced diet.

#### 2.2.3. Clinical Observations and Appearance

The appearance, movement and behaviour of all mice remained normal throughout the 21-day experimental period.

#### 2.2.4. Bodyweight and Food Consumption

**Figure 1:**
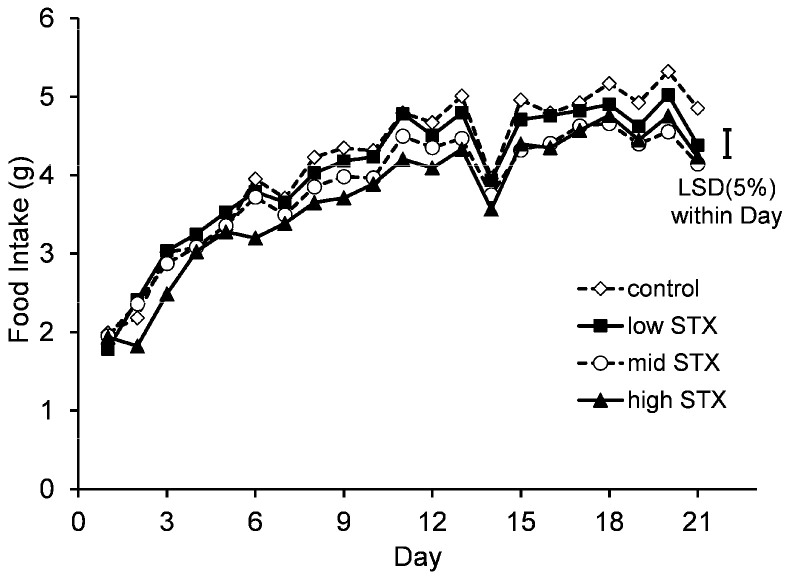
Temporal trend in food intake of mice fed control (^..^◊^..^), low STX (-■-), mid STX (^..^○^..^) or high STX (-▲-) diets (1.14, 2.28 and 3.48 µg STX.2HCl/g, respectively) over the 21-day feeding study. The error bar denotes the Fisher’s unprotected least significant differences at the 5% level (LSD (5%)).

Statistical analysis of the daily food intake data showed that there was no evidence of an interaction between gender and treatment (gender.treatment.day, *p* = 0.940; gender.treatment, *p* = 0.198). A graph could therefore be created for the temporal treatment effect pooled over gender (Figure 1).

The food consumption of all mice was low at the start of the study as they adapted to the meal time feeding protocol. The dips in food intake on days 14 and 21 were observed in all treatment groups and coincided with the days that motor coordination, blood pressure, heart rate and grip strength were measured. Although still present, this observed dip in food intake was less marked on day 7 when only motor coordination and grip strength were measured. This demonstrated that the measurement of blood pressure and heart rate was the major driver of this disruption of mouse feeding. The statistical analysis of the pooled data showed that although feeding of mice in all treatment groups was good, the presence of STX did cause a dose-dependent statistical effect on food intake. Mice fed a high STX diet ate a statistically lower amount of food than mice fed a control diet on 16 of the 21 days. Similarly, mice fed with a mid and low STX diet ate a statistically lower amount of food than mice fed a control diet on 9 and 1 of the 21 days, respectively.

**Figure 2:**
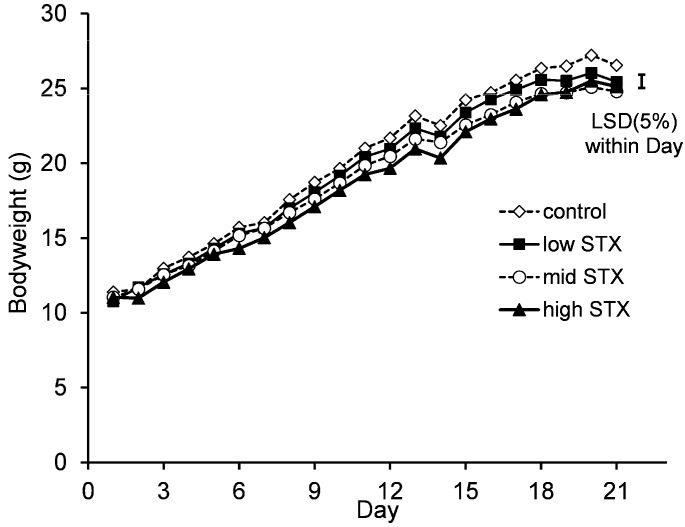
Temporal trend in bodyweights of mice fed control (^..^◊^..^), low STX (-■-), mid STX (^..^○^..^) or high STX (-▲-) diets (1.14, 2.28 and 3.48 µg STX.2HCl/g, respectively) over the 21-day feeding study. The error bar denotes the Fisher’s unprotected least significant differences at the 5% level (LSD (5%)).

Statistical analysis of the bodyweights of mice showed no evidence of an interaction between gender and treatment (gender.treatment.day, *p* = 0.918; gender.treatment, *p* = 0.393). A graph could therefore be created for the temporal treatment effect pooled over gender (Figure 2).

This bodyweight data showed that all mice gained weight over the experimental period but mice fed a high, mid and low STX diet gained a lower amount of weight on average than mice fed a control diet on 17, 13 and 3 of the 21 days of the study, respectively.

#### 2.2.5. Motor Coordination

**Figure 3:**
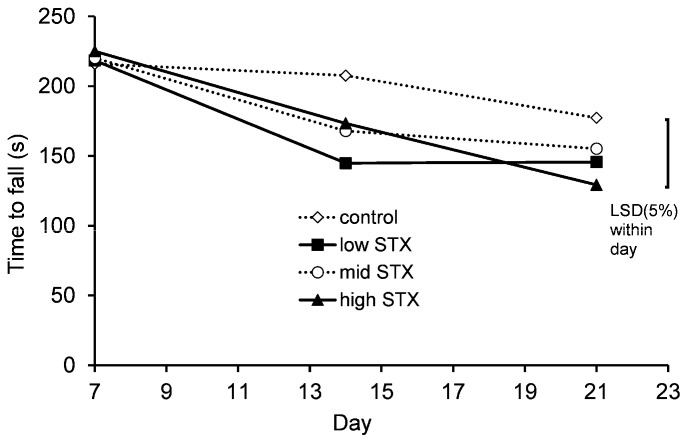
Temporal trend in motor coordination (time to fall) of mice fed control (^..^◊^..^), low STX (-■-), mid STX (^..^○^..^) or high STX (-▲-) diets (1.14, 2.28 and 3.48 µg STX.2HCl/g, respectively) on days 7 and 14 of the feeding study. The error bar denotes the Fisher’s unprotected least significant differences at the 5% level (LSD (5%)).

Motor coordination of all mice was analysed on days 7, 14 and 21 using an accelerating rotarod. Statistical analysis of the motor coordination data showed no evidence of an interaction between gender and treatment (gender.treatment.day, *p* = 0.113; gender.treatment, *p* = 0.761). A graph could therefore be created for the temporal treatment effect pooled over gender (Figure 3). There were no statistically significant treatment effects on the motor coordination of mice.

#### 2.2.6. Grip Strength

**Figure 4:**
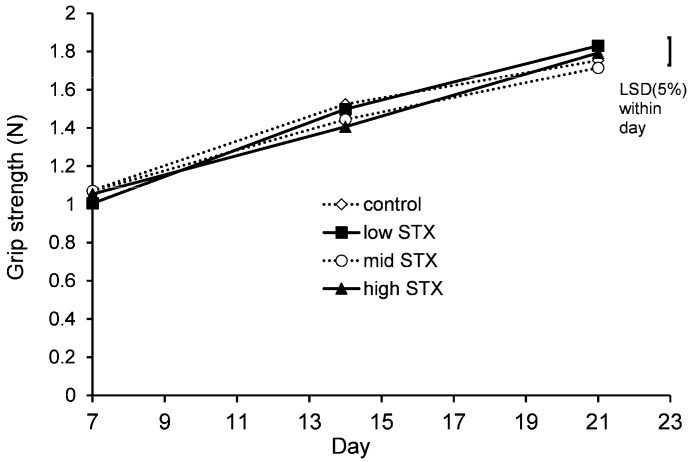
Temporal trend in grip strength of mice fed control (^..^◊^..^), low STX (-■-), mid STX (^..^○^..^) or high STX (-▲-) diets (1.14, 2.28 and 3.48 µg STX.2HCl/g, respectively) on days 7, 14 and 21 of the feeding study. The error bar denotes the Fisher’s unprotected least significant differences at the 5% level (LSD (5%)).

The grip strength of all mice was analysed on days 7, 14 and 21. Statistical analysis of the grip strength data showed no evidence of an interaction between gender and treatment (gender.treatment.day, *p* = 0.574; gender.treatment, *p* = 0.334). A graph could therefore be created for the temporal treatment effect pooled over gender (Figure 4). The data showed no statistically significant treatment effects on the grip strength of mice.

#### 2.2.7. Blood Pressure and Heart Rate

The blood pressure and heart rate were measured in all mice on days 14 and 21 (Table 3). Measurements were not possible prior to day 14 as the mice were too small to fit into the blood pressure analysis system. There were no statistically significant differences observed in systolic blood pressure, diastolic blood pressure or heart rate between any of the treatment groups.

#### 2.2.8. Haematological and Serum Biochemical Data

The haematological data of blood samples collected on day 21 are presented in Table 4. For the haemoglobin level (HB), a small, but dose-dependent, decrease was seen for the male mice with the means of the mid STX and high STX treatment groups being statistically significantly different from that of the control group. However, this treatment group effect was less compelling for the female mice, with no statistically significant differences between the control group and any of the three STX treatment groups, making it unlikely that the differences seen in the male mice were of any significance. The mean white blood cell counts were highest in the control group for both genders, although it was only the values for male mice which showed a statistically significant difference. Furthermore, there was no STX dose-dependent effect for either gender, meaning that these results are unlikely to be toxicologically significant.

The serum biochemical data of blood samples collected on day 21 are presented in Table 5. The aspartate aminotransferase (AST) and alanine aminotransferase (ALT) data were log transformed to stabilise the variance. For female mice, the log ALT is statistically significantly higher for the control than for any of the STX fed mice, but this effect did not show a dose-dependent trend. Furthermore, male mice fed a control diet had the lowest log ALT of any of the treatment groups meaning that this observation is highly unlikely to be of toxicological significance. Creatinine was also statistically significantly higher in the control female group in comparison to the treatment groups fed low STX and mid STX diets. However, no difference was seen between the female control and STX high dose mice, and no effect was seen in the male mice, pointing to random chance rather than any effect of treatment. For the male mice, the log AST was statistically higher in the STX high dose group in comparison to the control, but since the trend in the female mice was the opposite, this is unlikely to be of any importance.

#### 2.2.9. Organ Weights

The organ weights of all mice, expressed as the percentage of bodyweight, are presented in Table 6. There was no strong statistical evidence of an overall treatment effect on relative organ weights. Neither the interaction between gender and treatment (*p* ≥ 0.065) or the main effect of treatment (*p* ≥ 0.081) was statistically significant at the 5% level for any of the organs expressed as percentage of bodyweight. When the pairwise comparisons within gender were examined (Table 6), some statistically significant differences between the means were observed. However, in each case, there was either no dose-dependency or the effect was only seen in one gender. It is therefore unlikely that any of these observations are of any toxicological significance.

#### 2.2.10. Histological Examination

No significant changes in tissues were visible on histological examination in mice from any of the treatment groups.

## 3. Discussion

In this experiment, using a meal time feeding protocol, male and female mice were fed daily dose rates of up to 699 and 730 µg STX.2HCl/kg bw/day, respectively, for 21 days, without developing any signs of toxicity (average of 715 µg STX.2HCl/kg bw/day). A small, but dose-dependent, effect of STX on the food intake of mice was detected. This may have been due to palatability of the diet to mice or it may have been due to post-digestional malaise. This will be explored further in a future experiment. None of the mice showed any behavioural or physiological effects, the serum biochemistry was normal, and in addition, there were no changes detected at histopathological examination of tissue samples. The NOAEL of STX.2HCl, administered using a meal time feeding protocol, is therefore greater than 715 µg STX.2HCl/kg bw. Taking into account the 100-fold safety factor to extrapolate from animal data to humans, this yields a human safe level equivalent of 7.2 µg STX.2HCl/kg. The dose rates used in this study far exceed the ARfD of 0.5 µg STX.2HCl/kg bw suggested by EFSA. To reach the human equivalent of the dose rates used in this study (7.2 µg STX.2HCl/kg bw), a 60 kg human would have to ingest 540 g of shellfish flesh contaminated with PSTs at the current regulatory limit (800 µg STX.2HCl/kg flesh). For a human weighing a more realistic 70 kg, this portion size increases to 630 g. The highest shellfish portion size proposed for use in risk assessment is that of 400 g by EFSA [7]. However, the FAO/IOC/WHO (2004) Committee noted that a portion size of 250 g would cover 97.5% of the consumers of most countries for which data were available [8].

Animal models, using the oral route of exposure, will reflect the potential human health risk of toxins in food. In this experiment, feeding was chosen as the method of oral administration rather than gavage. This was for a number of reasons. Tingling and numbness of the lips and mouth have been reported as amongst the first symptoms of human PSP cases which demonstrates that local absorption of the toxins through the buccal mucous membranes occurs with oral ingestion of these toxins [29]; this process would be bypassed by using gavage administration. Additionally, numerous studies have shown that gavage dosing overestimates the toxicity of shellfish toxins [21,30]. This is likely due to the consistency of mouse stomach contents as, unlike humans, the stomach contents of rodents are semi-solid which could allow part of the liquid gavage dose to flow around them to be rapidly absorbed by the duodenum [19,31]. In contrast, if the test compound is incorporated with a solid matrix, it mixes with the existing stomach contents. Furthermore, a study by Craig and Elliott [32] using radiolabelled protein showed that 38% of mice dosed by gavage (27/71) did not receive their liquid dose correctly. Mice which had received an incorrect gavage dose showed no changes in behaviour meaning that they could not be distinguished from those which had been successfully dosed. This was attributed to occasional spillover/regurgitation of the dosing material into the lungs and led the authors to conclude that “the common method of gavage feeding mice to assess absorption of orally ingested material can lead to artifacts not seen when the same agent is consumed under more natural circumstances” [32].

As an alternative to gavage, we incorporated STX.2HCl into the diet of mice. Although laced cream cheese has been used successfully in acute toxicity testing [27], this was not suitable for a feeding study as, over time, mice tend to start refusing to eat the dose, an effect which would have a major impact on the study. As an alternative, STX was incorporated into the normal diet of mice, a method which worked well. Mice with unlimited access to STX-containing diet ingested a dose rate of up to 620 µg STX.2HCl/kg bw/day for four days without any symptoms of toxicity. Given that the oral LD_50_ for STX.2HCl in mice is 1063 µg/kg bw and the NOAEL is 473 µg/kg bw [21], this lack of toxicity was surprising. However, a one-off bolus dose, such as that used in the acute toxicity work, is a vastly different scenario to the constant exposure to small quantities of STX over a long period of time as would occur with the unrestricted feeding protocol. The feeding behaviour of mice is one of many bouts of feeding (grazing), with 70% of food consumed in the dark and 30% during the light [33,34]. This pattern is totally different to human feeding behaviour which is that of meal times and which would result in the ingestion of STX over a much shorter time period. To replicate this scenario, a feeding protocol was developed whereby mice were fed for two 1 h feeding periods per day. Mice quickly adapted to this change in feeding regime and after a training period of three days their daily food intake was normal. Preliminary testing of STX.2HCl using this protocol also showed a surprising lack of toxicity. It has been suggested by other authors [35] that the ingestion of PSTs mixed with 150 mg of cream cheese may influence toxicity due to the high fat content of cream cheese. To ensure that the lack of observed toxicity with feeding was not simply a matrix effect (cream cheese versus mouse food), one mouse was fed meals of diet laced with STX, while another was fed meals of control diet along with STX-laced cream cheese. Both methods delivered the same high dose rate of STX.2HCl (400 µg/kg bw twice a day) and both showed no symptoms of toxicity. In contrast, a mouse with unrestricted access to control diet developed severe symptoms of toxicity after just one bolus dose of 400 µg STX.2HCl/kg bw in cream cheese. This difference in toxicity must therefore be caused by the different quantity of food in the stomach of mice at the time of STX ingestion. With unlimited access to food, the stomach of a mouse would be relatively empty in the morning when the STX-laced cream cheese was consumed as a bolus dose. In contrast, the mice fed meals of STX-laced diet consumed half of their daily food intake along with the STX, resulting in the stomach containing considerable solid material. The quantity of matrix (food) in the stomach of mice is therefore impacting the absorption of STX and thus influencing toxicity. Relating this to a human scenario, STX in pure form cannot be eaten, meaning that it is not possible to ingest STX on an empty stomach. Even the standard portion size of 100 g determined by EFSA would result in the ingestion of a significant amount of matrix (food) along with the STX. In this experiment, using the meal time feeding protocol, mice were fed daily dose rates of up to 730 µg STX.2HCl/kg bw/day for 21 days without developing any signs of toxicity.

The high dose rate used in our feeding study, which induced no adverse effects, is at odds with a study by Arnich and Thébault [36] who, on the basis of the quantitative modelling of human PSP cases, proposed a lowest observed adverse effect level (LOAEL) of 0.37 µg STX.2HCl eq/kg bw. After applying the 100-fold safety factor for extrapolation from animals to human, the NOAEL determined in our feeding study was almost 20 times lower than this figure. However, as discussed earlier, the STX.2HCl concentrations in left-over food, the quantity of shellfish eaten and the bodyweight of the human are required to effectively interpret PSP cases. The Arnich and Thébault [36] model was based on 13 outbreaks of PSP which were often missing some of these crucial pieces of information. Furthermore, in all but one of the outbreaks, the PSP toxin analysis was conducted by mouse bioassay (MBA) [37]. This MBA was developed in the 1930s, whereby the relationship between the i.p. dose of pure STX.2HCl and the death time of mice was established to yield a dose-death time curve [38]. This curve was then used to convert the death time of mice injected with shellfish samples into STX.2HCl equivalents. However, PSTs are comprised of a large number of analogues and it has been demonstrated that the different analogues can have a dose-death time curve which is not consistent with that of STX [28]. The MBA approach therefore does not accurately determine the concentrations of PSTs. With the advanced analytical tools now available, along with the push for better information to be collected from patients presenting at hospitals with PSP, better data to input into the model should be available in the future. Another study which appears to contradict our results is that of Boente-Juncal et al. [39] who administered combinations of STX and tetrodotoxin (TTX) by gavage for 28 days. The dose rates used were very low, 44 µg TTX/kg bw along with 5.3, 17 or 54 µg STX.2HCl/kg bw. Surprisingly, one out of the four mice dosed at the lowest dose and two out of five mice dosed at the highest dose rate died. Each of these deaths were described as sudden convulsions and rapid death which perhaps could be attributable to the inherent issues with gavage dosing. Given the mouse deaths, symptoms of toxicity would have been expected in the surviving mice, but all survivors showed no symptoms of toxicity and gained weight normally. The mechanism of action for both STX and TTX is via voltage-gated sodium channels and the toxicities of the two toxins are additive when administered orally [21], resulting in the recommendation that TTX should be added to the PSTs. For this reason, it is valid to add the STX and TTX dose rates given in the Boente-Juncal et al. (2020) study [39] to allow a comparison with those administered in our study. The combined STX and TTX dose rates administered by gavage were 56.6, 68.3 and 105.3 µg STX.2HCl eq/kg bw. In comparison, the STX.2HCl dose rate administered to mice using the meal time feeding protocol in our study was 715 µg STX.2HCl eq/kg bw, seven times higher and with no adverse effects observed.

This feeding study showed no evidence of a cumulative effect of STX in mice, and on the basis of the high dose rates of STX.2HCl given using the meal time feeding protocol, designed to replicate human feeding behaviour, the current regulatory limit for PSTs seems adequate to protect human health. For the determination of this regulatory limit, the PSP cases considered by EFSA were all from the ingestion of non-commercial shellfish samples. Regulation of commercial samples has led to rigorous routine monitoring programmes and it was recognized by the working group convened to “assess the advice from the joint FAO/WHO/IOC Ad Hoc expert consultation on biotoxins and bivalve molluscs” that using the current limit of 800 µg STX.2HCl eq/kg shellfish flesh has resulted in no human illnesses from commercially harvested shellfish for 50 years [11]. This observation, as in our experiment, indicates that the current regulatory level for PSTs is fit for purpose. Since no toxicity was observed in the study, the true NOAEL exceeds the highest dose administered. To determine the true NOAEL, further work, using higher dose rates, would be required. This study highlighted that the feeding regime has a big influence on the toxicity of STX.2HCl. Further work is planned to better define this difference as it is possible that the current methods to determine acute oral toxicity for use in the setting of regulatory limits is overestimating the risk of these toxins to human health. It would be interesting to investigate whether the impact of feeding regime on toxicity is confined to just STX or whether it also occurs with other toxin classes.

## 4. Materials and Methods

### 4.1. Purity Assessment of Saxitoxin

STX was supplied by the Cawthron Institute (Nelson, NZ) and calibrated against certified reference material from the National Research Council of Canada (NRC) using HPLC-UV at 210 nm and a method adapted from Rourke et al. [40]. Ion pairing chromatography was performed on a Zorbax Bonus RP 4.6 × 150 mm 3.5 µm column under isocratic conditions over 30 min at 1 mL/min with 11 mM heptane sulfonate, 16.5 mM phosphoric acid 11.5% acetonitrile adjusted to pH 7.1 with ammonium hydroxide. Additionally, trace concentrations of other PSTs were quantified using LC-MS/MS [41], showing the material to contain 99.8% STX, 0.16% decarbamoylSTX and 0.05% neoSTX. The STX material was stored at 4 °C as a stock solution in 3 mM HCl at a concentration of 10.91 mg/mL STX.2HCl. For preparation of mouse diet, working solutions were prepared gravimetrically using 3 mM HCl as the diluting solvent.

### 4.2. Animals

Swiss albino mice were used for all experimental work. Females were used for the preliminary studies; however, for the feeding study, mice of both genders were used. Mice were individually caged in a temperature-controlled room (21 ± 1 °C) with a 12-h light–dark cycle and with unrestricted access to water. During the feeding study, a housing arrangement was used where the boxes, each containing an individual mouse, were randomised for columns and rows such that the eight combinations of treatment group and gender occurred exactly once along each row (forming the experimental replicate) and no more than once down each column.

### 4.3. Preparation of Mouse Diets

Mouse diets were based on Teklad Global 2016 mouse food pellets (Harlan UK, Bicester, UK) ground to a fine flour using a cyclone sample mill (Udy Corporation, Fort Collins, CO, USA). To check for STX homogeneity in the diet, a test batch was prepared by mixing ground mouse food (50 g) with water (45 mL) containing STX.2HCl (13.5 µg). This mixture was well combined and seven cookie-shaped portions of diet (approximately 2.5 × 2.5 × 1 cm) were prepared and dried in a fan oven (50 °C for 24 h). After the drying process had been completed, three different areas of one portion of the STX-laced diet were taken and ground using a mortar and pestle. The STX concentration was measured by the analysis of duplicate samples by LC-MS/MS. Since analysis showed that homogeneity was a problem in this diet, a further test batch of STX-laced diet was prepared by taking ground mouse food (25 g) and mixing this with water (20 mL) containing STX.2HCl (87.5 µg). This mixture was well-combined and 25 small cookie-shaped portions of diet (1.5 cm diameter and 0.6 cm thickness) were prepared and dried in a fan oven at a lower temperature and for a shorter time (30 °C for 16 h) compared to the initial batch. The resulting STX-laced diet was extracted and analysed (as detailed in Section 4.4), which showed that the homogeneity issue had been resolved. To check the stability of STX, portions of the laced diet were stored at both 4 °C and 20 °C. Two samples of the stored diet were taken on days 1, 2 and 5, and processed as detailed in Section 4.4. Each sample was analysed in triplicate.

During the feeding trial, 50 g batches of STX-laced diet were prepared as above, resulting in 50 small portions of diet. Each batch was weighed at the end of the drying process so that the moisture contents could be calculated. The average moisture content was 14.1% for the control diet, 15.4% for the low dose STX diet, 13.3% for the mid dose STX diet and 14.2% for the high dose STX diet. To determine the concentrations of the three different STX diets used in the feeding trial, two samples of each treatment diet were ground using a mortar and pestle and then analysed in quadruplicate using the methods described in Section 4.4. Prepared diets were kept at 4 °C until use and were prepared at least every three days during the feeding study.

### 4.4. LC-MS Analysis

To determine the STX concentration in laced diet, samples were taken (200 ± 5 mg) and extracted with 0.1 M HCl (10 mL) by placing them in a boiling water bath (5 min). The extracts were then cooled in an ice bath (5 min), briefly mixed on a vortex mixer and then centrifuged (17,000× *g* for 5 min). An aliquot of the resulting extract (10 µL) was added to 80% acetonitrile with 0.25% acetic acid (490 µL) in a polypropylene autosampler vial and analysed by UHPLC-MS/MS based on the method of Turner et al. [42], using a 6500+ QTRAP tandem quadrupole mass spectrometer coupled to an Exion LC liquid chromatography separations system, with high pH compatibility conversion composed of a multiplate autosampler, binary solvent pumps with low pressure gradient proportioning valve solvent selection, and a column oven with 6-port 2-position column selection valve. Curtain gas was 25 psi, ion source gas 1 was 50 psi, ion source gas 2 was 50 psi, ionspray voltage was −4500 in negative mode, and 5500 V in positive mode, the temperature was 500 °C, and declustering potential was −30 V in negative mode and 30 V in positive mode. Scheduled MRM mode was used for data acquisition with STX monitored in positive ion mode with MRM transitions 300.141 > 204.088 (CE 34 V) and 300.141 > 138.066 (CE 36 V).

### 4.5. Preliminary Work and Palatability Studies

To determine an appropriate STX dose rate, pairs of mice were housed together and given unrestricted access to STX-laced diet. Food consumption and bodyweight were measured daily to allow the calculation of the STX dose rate ingested. Initially, a pair of mice was fed a diet containing 2.43 µg STX.2HCl/g for 14 days, which delivered an average dose rate of 476 µg STX.2HCl/kg bw/day. In a further trial, a pair of mice was fed a diet containing 2.95 µg STX.2HCl/g for four days, which delivered an average dose rate of 620 µg STX.2HCl/kg bw/day.

To develop experimental protocols to replicate human feeding behaviour, one individually caged mouse was given unrestricted access to control diet and another was fed the same diet between only 8–10 a.m. and 3–5 p.m. for eight days. Daily food intake and bodyweight were measured to allow g food consumed/g bw to be calculated.

To ensure that the administration of STX in the laced diet was not influencing toxicity, three weanling mice were taken and individually caged. Mouse 1 had unrestricted access to control diet and was trained to eat small quantities of cream cheese following a method previously described [21]. Mouse 2 was trained in the meal time feeding protocol using control diet as well as being trained to eat small quantities of cream cheese, and mouse 3 was trained in the meal time feeding protocol using control diet. After four days of training, mouse 1 continued to have unrestricted access to control diet but was fed cream cheese (150 mg) laced with STX. Mouse 2 was fed control diet at meal times along with cream cheese (150 mg) laced with STX half-way through each of its two daily feeding periods, and mouse 3 was fed STX-laced diet at meal times. Mouse 1 ingested 400 µg STX.2HCl/kg bw as a one-off bolus dose, and both mouse 2 and 3 ingested 400 µg STX.2HCl/kg bw at each of their twice daily feeding periods. The mice were observed closely for any signs of toxicity throughout the day.

### 4.6. Sub-Acute 21-Day Feeding Trial

Sixty weanling mice (30 female and 30 male) were individually caged and trained in the meal time feeding protocol. To allow a more gradual adjustment to the change in feeding regime, mice were given access to food between 9 a.m. and 5 p.m. for one day, and then for the following three days were fed between 9–10 a.m. and 3.30–4.30 p.m. This period of training was completed with control diet. On each day of the training period, the amount of food consumed by each mouse at each meal time was measured. On each of the two days before the start of the study, mice were trained on an accelerating rotarod (Rotamex 4/8, Columbus Instruments International, Columbus, OH, USA) by placing them on the accelerating rod (13–79 rpm over 12 min) and recording the time to fall. Each mouse was given two attempts per day. Mice which did not adapt to the meal time feeding protocol or those which performed poorly during training on the accelerating rotarod were excluded from the study. From those mice remaining, 20 females and 20 males were selected and randomly assigned to treatment groups. Four treatment groups, each containing five female and five male mice (individually caged) were fed between 9–10 a.m. and 3.30–4.30 p.m. for 21 days with the following diets. Group 1—control diet, Group 2—low-dose STX-laced diet (1.14 µg STX.2HCl/g), Group 3—mid-dose STX-laced diet (2.28 µg STX.2HCl/g), and Group 4—high-dose STX-laced diet (3.39 µg STX.2HCl/g). All animals had unrestricted access to water and food consumption was measured after each twice daily meal time. In addition, after each daily afternoon feed, the bodyweight of each mouse was recorded and posture/appearance noted. On days 14 and 21, heart rate and blood pressure was determined for each mouse using a Visitech Systems BP-2000 blood pressure analysis system (Visitech Systems, Apex, NC, USA). On days 0, 7, 14 and 21, motor coordination was evaluated using the accelerating rotarod as described above and grip strength was measured using a grip strength meter (MK3805S, Muromachi, Tokyo, Japan). On each measurement day, the mice were given two trials on the rotarod and grip strength meter, with the results being averaged. At the end of the 21-day feeding period, mice were euthanised by CO_2_ inhalation and blood samples collected by heart puncture with heparin as the anticoagulant. Haematocrit values (HCT), haemoglobin levels (HB), mean corpuscular volumes (MCV), mean corpuscular haemoglobin (MCH), mean corpuscular haemoglobin concentrations (MCHC), and red and white blood cell counts were measured in whole blood. In addition, plasma was analysed for activities of aspartate aminotransferase (AST), alanine aminotransferase (ALT), and for levels of urea, total protein (TP), albumin (ALB), globulin, sodium, potassium, chloride and creatinine (CRN) (IDEXX laboratories, Hamilton, NZ). At necropsy, any macroscopic changes observed were recorded and the weights of brain, heart, kidneys, liver and spleen measured and expressed as percentages of bodyweight. These tissues, together with adrenals, lungs, pancreas, gastrocnemius, jejunum (3 mm section), ovary/uterus or testes, spinal cord (3 × 2 mm sections), stomach (washed), thymus and urinary bladder were fixed in 4% buffered formaldehyde and routinely processed for histological examination. All samples were assessed by the same pathologist who was blinded to the treatment group of the samples.

### 4.7. Statistical Analysis

The bodyweight, food consumption, motor coordination, grip strength, blood pressure and heart rate data were analysed using repeated measures formulated as linear mixed effects models and fitted using residual maximum likelihood (REML). The random model comprised of random effects for replicate (i.e., the row in the housing arrangement) and mouse. The basic fixed model comprised of effects for treatment group, day, gender and all two- and three-way interactions. In the analyses of bodyweight and food consumption date, pre-treatment (i.e., day 0) bodyweight was also included as a fixed effect covariate, and in the analyses of motor coordination and grip strength, the pre-treatment measurement on day 0 was included as a fixed effect covariate. Repeated measures on the same mouse over time were assumed to be correlated with a first-order autoregressive structure.

The haematological, serum biochemical and organ weight data were analysed using linear mixed effects models fitted using REML with random effects for replicate and fixed effects for kill day, treatment group, gender and the treatment group by gender interaction. Serum AST and ALT were log transformed to stabilise the variance.

In all analyses, the variance components were constrained to be positive, residual diagnostic plots were inspected for evidence of departures from the residual assumptions of normality and constant variance, a 5% significance level was used when assessing the fixed effects, and Fisher’s unprotected least significant differences at the 5% level (LSD (5%)) were used to compare means. Statistical analyses were performed using Genstat 19th Edition (VSN International, Hemel Hempstead, UK).

## Figures and Tables

**Table 1:** Saxitoxin concentrations in mouse diet stored at different temperatures and for different time periods.

Sample	Concentration (µg STX.2HCl eq/g) ^1^	Recovery ^2^
Day 1 Control	3.30 ± 0.24	95%
Day 2 20 °C	3.30 ± 0.48	95%
Day 2 4 °C	3.26 ± 0.36	94%
Day 5 20 °C	3.33 ± 0.24	96%
Day 5 4 °C	3.26 ± 0.45	94%

^1^ ± 95% confidence interval, ^2^ Compared to theoretical maximum of 3.48 µg STX.2HCl eq/g.

**Table 2:** Saxitoxin concentrations in mouse diet for the three different treatments.

Treatment	Measured Concentration (µg STX.2HCl eq/g) ^1^	Theoretical Concentration (µg STX.2HCl eq/g)	Recovery ^2^
Low STX	1.12 ± 0.20	1.15	97%
Mid STX	2.34 ± 0.41	2.27	103%
High STX	3.19 ± 0.56	3.41	94%

^1^ ± 95% confidence interval, ^2^ Compared to theoretical.

**Table 3:** Heart rate and blood pressure of mice fed control, low STX, mid STX or high STX diets (1.14, 2.28 and 3.48 µg STX.2HCl/g, respectively) on days 14 and 21 of the feeding study.

	Heart Rate ^1^ (BPM)		Systolic BP ^1^ (mmHg)		Diastolic BP ^1^ (mmHg)	
	Day 14	Day 21	Day 14	Day 21	Day 14	Day 21
*Females*						
control	729 ± 16.7 ^a^	710 ± 16.7 ^a^	112.6 ± 6.89 ^a^	119.0 ± 6.89 ^a^	45.8 ± 4.20 ^a^	52.0 ± 4.20 ^a^
low	732 ± 16.7 ^a^	679 ± 16.7 ^a^	110.2 ± 6.89 ^a^	118.4 ± 6.89 ^a^	54.4 ± 4.20 ^a^	57.2 ± 4.20 ^a^
mid	753 ± 16.7 ^a^	690 ± 16.7 ^a^	105.4 ± 6.89 ^a^	111.0 ± 6.89 ^a^	52.6 ± 4.20 ^a^	49.4 ± 4.20 ^a^
high	754 ± 16.7 ^a^	699 ± 16.7 ^a^	99.4 ± 6.89 ^a^	106.0 ± 6.89 ^a^	43.6 ± 4.20 ^a^	52.8 ± 4.20 ^a^
*Males*						
control	709. ± 16.7 ^a^	704 ± 16.7 ^a^	108.4 ± 6.89 ^a^	111.8 ± 6.89 ^a^	48.2 ± 4.20 ^a^	48.0 ± 4.20 ^a^
low	701. ± 16.7 ^a^	692 ± 16.7 ^a^	105.2 ± 6.89 ^a^	119.4 ± 6.89 ^a^	41.0 ± 4.20 ^a^	49.2 ± 4.20 ^a^
mid	753 ± 16.7 ^a^	695 ± 16.7 ^a^	101.4 ± 6.89 ^a^	116.2 ± 6.89 ^a^	42.8 ± 4.20 ^a^	50.8 ± 4.20 ^a^
high	729 ± 16.7 ^a^	680 ± 16.7 ^a^	112.6 ± 6.89 ^a^	116.0 ± 6.89 ^a^	50.8 ± 4.20 ^a^	50.8 ± 4.20 ^a^

^1^ Values are means ± standard error of the mean (n = 5). Fisher’s unprotected least significant differences were used to compare the treatment means within each sex. Two means that have no letter in common are statistically different at the 5% level.

**Table 4:** Haematology data of mice fed control, low STX, mid STX or high STX diets (1.14, 2.28 and 3.48 µg STX.2HCl/g, respectively) for 21 days.

Item	Control ^1^	Low STX ^1^	Mid STX ^1^	High STX ^1^
*Females*				
HCT (L/L)	0.45 ± 0.01 ^a^	0.47 ± 0.01 ^a^	0.46 ± 0.01 ^a^	0.44 ± 0.01 ^a^
HB (g/L)	136 ± 2.9 ^ab^	141 ± 2.9 ^b^	138 ± 2.9 ^ab^	132 ± 2.9 ^a^
RBC (×10^12^/L)	8.29 ± 0.21 ^a^	8.75 ± 0.21 ^a^	8.61 ± 0.21 ^a^	8.31 ± 0.21 ^a^
MCV (fL)	54.5 ± 0.70 ^a^	53.9 ± 0.70 ^a^	53.9 ± 0.70 ^a^	53.3 ± 0.70 ^a^
MCH (pg)	16.4 ± 0.18 ^a^	16.2 ± 0.18 ^a^	16.0 ± 0.18 ^a^	16.0 ± 0.18 ^a^
MCHC (g/L)	303 ± 3.1 ^a^	299 ± 3.1 ^a^	297 ± 3.1 ^a^	299 ± 3.1 ^a^
WBC (×10^9^/L)	6.51 ± 0.82 ^a^	5.51 ± 0.82 ^a^	4.55 ± 0.82 ^a^	5.25 ± 0.82 ^a^
Neutrophil (%)	9.56 ± 2.73 ^a^	11.24 ± 2.73 ^a^	6.76 ± 2.73 ^a^	10.56 ± 2.73 ^a^
Lymphocyte (%)	87.3 ± 3.84 ^a^	85.3 ± 3.84 ^a^	91.9 ± 3.84 ^a^	85.7 ± 3.84 ^a^
Monocyte (%)	1.29 ± 0.95 ^a^	2.71 ± 0.95 ^a^	0.89 ± 0.95 ^a^	1.89 ± 0.95 ^a^
Eosinophil (%)	0.89 ± 0.586 ^a^	0.91 ± 0.586 ^a^	0.29 ± 0.586 ^a^	1.29 ± 0.586 ^a^
*Males*				
HCT (L/L)	0.46 ± 0.01 ^a^	0.47 ± 0.01 ^a^	0.46 ± 0.01 ^a^	0.45 ± 0.01 ^a^
HB (g/L)	137 ± 2.9^b^	136 ± 2.9 ^b^	135 ± 2.9 ^ab^	128 ± 2.9 ^a^
RBC (×10^12^/L)	8.58 ± 0.21 ^a^	8.57 ± 0.21 ^a^	8.57 ± 0.21 ^a^	8.12 ± 0.21 ^a^
MCV (fL)	54.0 ± 0.70 ^a^	54.7 ± 0.70 ^a^	53.5 ± 0.70 ^a^	54.8 ± 0.70 ^a^
MCH (pg)	16.0 ± 0.18 ^a^	16.0 ± 0.18 ^a^	16.0 ± 0.18 ^a^	16.0 ± 0.18 ^a^
MCHC (g/L)	295 ± 3.1 ^a^	291 ± 3.1 ^a^	294 ± 3.1 ^a^	288 ± 3.1 ^a^
WBC (×10^9^/L)	7.36 ± 0.82 ^b^	4.51 ± 0.82 ^a^	5.25 ± 0.82 ^a^	5.62 ± 0.82 ^ab^
Neutrophil (%)	13.85 ± 2.73 ^b^	13.44 ± 2.73 ^b^	11.64 ± 2.73 ^ab^	5.95 ± 2.73 ^a^
Lymphocyte (%)	82.4 ± 3.84 ^a^	84.5 ± 3.84 ^a^	85.3 ± 3.84 ^a^	90.9 ± 3.84 ^a^
Monocyte (%)	2.40 ± 0.95 ^a^	1.31 ± 0.95 ^a^	1.71 ± 0.95 ^a^	2.41 ± 0.95 ^a^
Eosinophil (%)	1.00 ± 0.586 ^a^	0.91 ± 0.586 ^a^	1.51 ± 0.586 ^a^	0.78 ± 0.586 ^a^

^1^ Values are means ± standard error of the mean (n = 5). Fisher’s unprotected least significant differences were used to compare the treatment means within each sex. Two means that have no letter in common are statistically different at the 5% level. HCT, haematocrit value; HB, haemoglobin level; RBC, red blood cells; MCV, mean corpuscular volume; MCH, mean corpuscular haemoglobin; MCHC, mean corpuscular haemoglobin concentration; WBC, white blood cells.

**Table 5:** Serum biochemical data of mice fed control, low STX, mid STX or high STX diets (1.14, 2.28 and 3.48 µg STX.2HCl/g, respectively) for 21 days.

Item	Control ^1^	Low STX ^1^	Mid STX ^1^	High STX ^1^
*Females*				
log AST (log IU/L)	5.81 ± 0.33 ^a^	5.27 ± 0.33 ^a^	5.45 ± 0.33 ^a^	5.46 ± 0.33 ^a^
log ALT (log IU/L)	5.25 ± 0.35 ^b^	3.65 ± 0.35 ^a^	4.01 ± 0.35 ^a^	3.85 ± 0.35 ^a^
Urea (mmol/L)	8.96 ± 2.12 ^a^	9.50 ± 2.12 ^a^	8.94 ± 2.12 ^a^	7.72 ± 2.12 ^a^
TP (g/L)	47.0 ± 1.7 ^a^	47.33 ± 1.7 ^a^	49.07 ± 1.7 ^a^	51.25 ± 1.7 ^a^
ALB (g/L)	28.6 ± 1.03 ^a^	28.0 ± 1.03 ^a^	29.0 ± 1.03 ^a^	30.6 ± 1.03 ^a^
Globulin (g/L)	19.8 ± 0.87 ^a^	19.5 ± 0.87 ^a^	20.5 ± 0.87 ^a^	21.3 ± 0.87 ^a^
CRN (µmol/L)	10.4 ± 1.04 ^b^	7.8 ± 1.04 ^a^	7.2 ± 1.04 ^a^	9.4 ± 1.04 ^ab^
A/G ratio	1.39 ± 0.03 ^a^	1.41 ± 0.03 ^a^	1.42 ± 0.03 ^a^	1.44 ± 0.03 ^a^
Na (mmol/L)	151 ± 1.3 ^a^	152 ± 1.3 ^a^	152 ± 1.3 ^a^	149 ± 1.3 ^a^
K (mmol/L)	8.07 ± 0.71 ^a^	7.81 ± 0.71 ^a^	7.53 ± 0.71 ^a^	8.35 ± 0.71 ^a^
Cl (mmol/L)	114.0 ± 1.02 ^a^	113.4 ± 1.02 ^a^	114.6 ± 1.02 ^a^	113.2 ± 1.02 ^a^
*Males*				
log AST (log IU/L)	4.72 ± 0.33 ^a^	5.27 ± 0.33 ^ab^	5.05 ± 0.33 ^ab^	5.75 ± 0.33 ^b^
log ALT (log IU/L)	3.54 ± 0.35 ^a^	3.58 ± 0.35 ^a^	3.68 ± 0.35 ^a^	3.79 ± 0.35 ^a^
Urea (mmol/L)	9.08 ± 2.12 ^a^	10.00 ± 2.12 ^a^	9.86 ± 2.12 ^a^	9.65 ± 2.12 ^a^
TP (g/L)	50.7 ± 1.7 ^a^	50.7 ± 1.7 ^a^	51.0 ± 1.7 ^a^	49.7 ± 1.7 ^a^
ALB (g/L)	28.2 ± 1.03 ^a^	28.6 ± 1.03 ^a^	28.8 ± 1.03 ^a^	28.2 ± 1.03 ^a^
Globulin (g/L)	22.5 ± 0.87 ^a^	22.7 ± 0.87 ^a^	22.3 ± 0.87 ^a^	21.7 ± 0.87 ^a^
CRN (µmol/L)	7.0 ± 1.04 ^a^	5.9 ± 1.04 ^a^	6.1 ± 1.04 ^a^	7.1 ± 1.04 ^a^
A/G ratio	1.24 ± 0.03 ^a^	1.26 ± 0.03 ^a^	1.28 ± 0.03 ^a^	1.30 ± 0.03 ^a^
Na (mmol/L)	152 ± 1.3 ^a^	151 ± 1.3 ^a^	151 ± 1.3 ^a^	152 ± 1.3 ^a^
K (mmol/L)	8.17 ± 0.71 ^a^	8.53 ± 0.71 ^a^	9.43 ± 0.71 ^a^	8.93 ± 0.71 ^a^
Cl (mmol/L)	111.4 ± 1.02 ^a^	113.0 ± 1.02 ^a^	112.0 ± 1.02 ^a^	114.0 ± 1.02 ^a^

^1^ Values are mean ± standard error of the mean (n = 5). Fisher’s unprotected least significant differences were used to compare the treatment means within each sex. Two means that have no letter in common are statistically different at the 5% level. AST, aspartate aminotransferase; ALT, alanine aminotransferase; TP, total protein; ALB, albumin; CRN, creatinine.

**Table 6:** Organ weights, expressed as percentage of bodyweight, for mice fed control, low STX, mid STX or high STX diets (1.14, 2.28 and 3.48 µg STX.2HCl/g, respectively) for 21 days.

Item	Control ^1^	Low STX ^1^	Mid STX ^1^	High STX ^1^
*Females*				
brain	1.86 ± 0.09 ^ab^	1.83 ± 0.09 ^a^	1.96 ± 0.09 ^ab^	2.01 ± 0.09 ^b^
heart	0.58 ± 0.03 ^a^	0.55 ± 0.03 ^a^	0.60 ± 0.03 ^a^	0.57 ± 0.03 ^a^
kidneys	1.34 ± 0.07 ^a^	1.24 ± 0.07 ^a^	1.30 ± 0.07 ^a^	1.30 ± 0.07 ^a^
liver	4.59 ± 0.10 ^a^	4.32 ± 0.10 ^a^	4.41 ± 0.10 ^a^	4.38 ± 0.10 ^a^
spleen	0.39 ± 0.03 ^a^	0.42 ± 0.03 ^a^	0.37 ± 0.03 ^a^	0.38 ± 0.03 ^a^
*Males*				
brain	1.73 ± 0.09 ^a^	1.72 ± 0.09 ^a^	1.74 ± 0.09 ^a^	1.83 ± 0.09 ^a^
heart	0.57 ± 0.03 ^a^	0.63 ± 0.03 ^b^	0.57 ± 0.03 ^ab^	0.55 ± 0.03 ^a^
kidneys	1.72 ± 0.07 ^b^	1.74 ± 0.07 ^b^	1.66 ± 0.07 ^ab^	1.59 ± 0.07 ^a^
liver	4.74 ± 0.10 ^a^	4.65 ± 0.10 ^a^	4.69 ± 0.10 ^a^	4.54 ± 0.10 ^a^
spleen	0.37 ± 0.03 ^b^	0.32 ± 0.03 ^ab^	0.34 ± 0.03 ^ab^	0.29 ± 0.03 ^a^

^1^ Values are mean ± standard error of the mean (n = 5). Fisher’s unprotected least significant differences were used to compare the treatment means within each sex. Two means that have no letter in common are statistically different at the 5% level.

## Data Availability

The data presented in this study are available on request from the corresponding author.
